# Wearable Device Photoplethysmography As a Viable Tool to Longitudinally Monitor Vasoconstriction Biomarkers for Predicting Vaso-Occlusive Crisis in Sickle Cell Disease: Feasibility and Validation Study

**DOI:** 10.2196/75465

**Published:** 2026-01-06

**Authors:** Payal Shah, Sabrina Sy, Mingjing Chen, Michael CK Khoo, Thomas D Coates, Saranya Veluswamy

**Affiliations:** 1 Division of Hematology, Cancer and Blood Disease Institute, Children's Hospital Los Angeles, University of Southern California, 4650 Sunset Boulevard, Los Angeles, CA, 90027, United States, 1 3233615507; 2 Alfred E. Mann Department of Biomedical Engineering, University of Southern California, Los Angeles, CA, United States

**Keywords:** sickle cell disease, wearable devices, microvascular blood flow, vasoconstriction, remote monitoring

## Abstract

**Background:**

Entrapment of sickled red blood cells in the microvasculature leads to sudden painful vaso-occlusive crises (VOCs) in sickle cell disease (SCD). This is potentially triggered by autonomic nervous system–mediated vasoconstriction in the microvasculature. Indeed, vasoconstriction biomarkers derived from a single night of laboratory-based fingertip photoplethysmography (PPG) recording were predictive of a higher frequency of future VOC in SCD. Noninvasive, remote, and longitudinal monitoring of autonomic vasoreactivity will facilitate the development of predictive biomarkers of imminent VOC.

**Objective:**

This study aimed to assess the feasibility and performance of a wearable wristband device to longitudinally monitor nocturnal peripheral autonomic vasoreactivity and to cross-validate the vasoconstriction parameters across the “gold-standard” finger sensor.

**Methods:**

A total of 12 patients with SCD and 6 healthy controls were recruited to wear a wristband device (Biostrap) with a PPG sensor on a nightly basis. For cross-validation studies, 50% (3/6) controls wore both the wristband and a sleep monitoring device (AliceNightOne) with a finger PPG sensor. We quantified autonomic vasoreactivity by processing PPG signals and deriving vasoconstriction parameters—magnitude of vasoconstriction (Mvasoc) and photoplethysmography amplitude coefficient of variation (PPGampCV). We performed a correlation analysis of the vasoconstriction parameters within each device to investigate whether Mvasoc and PPGampCV can be used as surrogate markers of vasoconstriction, and then cross-validated the PPGampCV across the wristband and finger PPG devices.

**Results:**

A total of 131 nocturnal PPG recordings were made with a wristband device (1‐19 nights per participant; patients with SCD: n=79, 60%; controls: n=52, 40%). A total of 9 nocturnal recordings (3 nights per participant) were made with both wristband and finger sensor devices. Longitudinal continuous PPG recordings were feasible with the wearable device, with significant within-night and night-to-night variability in vasoconstriction parameters, suggesting dynamic changes in autonomic vasoreactivity. Mvasoc and PPGampCV significantly correlated within devices—the maximum overnight correlation was 0.82 (*P*<.001) for the finger sensor and 0.69 (*P*<.001) for the wristband sensor, suggesting that PPGampCV can serve as a surrogate for Mvasoc. Cross-validation analysis of PPGampCV across wristband and fingertip sensors showed statistically significant correlations on all 9 nights (overnight correlation coefficient ranging from 0.24‐0.7), with some nightly segments of PPGampCV showing very strong correlation across devices.

**Conclusions:**

Wearable wristband devices are feasible tools for the collection of continuous PPG measurements and vasoconstriction parameters, which serve as objective markers of autonomic vasoreactivity in users with and without SCD. We have optimized the methods of quantifying vasoconstriction from wearable device PPG signals, and cross-validated them with standardized sensors. These findings enable large-scale, real-time monitoring of autonomic vasoreactivity along with pain outcomes for the development of vasoconstriction parameters as biomarkers imminent VOC in patients with SCD. This biomarker also has the potential to impact other diseases involving autonomic vascular dysregulation.

## Introduction

Vaso-occlusive crisis (VOC) due to obstruction of microvascular blood flow by sickled red blood cells is the prime reason for the morbidity and mortality in sickle cell disease (SCD) [1]. Peripheral vasoconstriction in the microvasculature due to dysregulated autonomic nervous system (ANS) responses and the subsequent reduced microvascular blood flow increases red blood cell transit time in the microvasculature, thus potentiating VOC [2-7]. Our recent work on the neurovascular physiology of SCD in humans has established that ANS-mediated decreases in microvascular blood flow, quantified by a novel vasoconstriction biomarker, strongly predict future VOC [8]. This novel vasoconstriction index, derived from the amplitude of finger photoplethysmography (PPG) signals, is a composite measure of the dynamic changes in peripheral blood flow occurring over time and is reflective of sympathetic neural inputs to the microvasculature. A single nocturnal measurement of this index in patients with SCD showed that a higher magnitude of vasoconstriction (Mvasoc) was predictive of increased hospitalizations for VOC in the upcoming years. However, day-to-day variations in autonomic vasoreactivity and their implications for imminent VOC risk remain unknown. All our previous measurements of peripheral vasoreactivity and ANS activity were performed in a well-controlled laboratory environment using standard finger PPG sensors to measure microvascular blood flow. Longitudinal remote monitoring of vasoconstriction events and ANS activity has a high potential for predicting and monitoring individualized pain episodes and is the necessary next step to develop nocturnal vasoconstriction indices as a predictor of imminent VOC.

Current wearable devices use optical PPG sensors to monitor heart rate variability and oxygen saturation. However, the raw PPG signals are often inaccessible before they go through automatic gain and processing, such that key information from the amplitude of the raw PPG signal is lost. Moreover, in the most commonly used commercial wearable devices, the PPG signal is measured from the wrist [9] or at the base of a finger [10,11]. These anatomic locations are not as highly perfused as the fingertip, and the PPG waveforms may be expected to be morphologically different from the fingertip due to differences in local vascular physiology and lower signal-to-noise ratios. On the other hand, wearable device PPG measurements are likely more reliable based on user compliance and practical considerations.

In this study, we established the use of PPG signals from a wrist-worn wearable device for real-time, remote monitoring of autonomic vasoreactivity and measurement of vasoconstriction events during sleep. The Biostrap EVO is a commercially available wrist-worn device (Biostrap LLC) that uses an optical PPG sensor to record standard continuous physiological data [12], including heart rate, computed respiratory activity, oxygen saturation (SpO2), and sleep and activity patterns [13]. Uniquely, our team was also able to access the raw PPG waveforms from the device, which is critical to quantify our key vasoconstriction parameters. The AliceNightOne (ANO) is a Food and Drug Administration–approved home sleep testing device (Koninklijke Philips N.V.), primarily designed to detect sleep apnea [14]. The 3-sensor device with a nasal cannula, finger PPG sensor, and chest belt detects nasal airflow pressure, SpO2, heart rate, respiratory effort, and body positioning. The ANO uses medical-grade “gold-standard” finger PPG probes to measure heart rate and oxygenation. The aims of this study were to

1. Assess the feasibility of wristband wearable devices to remotely monitor nocturnal peripheral autonomic vasoreactivity
2. Optimize the measurement of vasoconstriction parameters from wearable device PPG in patients with SCD and healthy controls.
3. Cross-validate the vasoconstriction parameters derived from a wearable wristband sensor (Biostrap) and a “gold-standard” finger sensor (ANO).

## Methods

### Ethical Considerations

The study was approved by the Institutional Review Board of the Children’s Hospital, Los Angeles, and all participants provided informed consent or assent to participate in the study. The study was conducted in accordance with the Declaration of Helsinki and the data was de-identified as per the Health Insurance Portability and Accountability Act (HIPAA), and subjects were compensated for their participation.

### Study Setup

Patients with SCD and healthy controls underwent autonomic monitoring with a wristband sensor to measure continuous PPG during sleep. Recordings were automatically triggered at sleep onset and paused for 5 minutes every hour to allow syncing of data wirelessly to a phone-based app. The raw PPG waveforms were made accessible from a cloud-based server, exported as .csv files, and selected for analyses based on the quality and completeness of the overnight recording. Raw signals were first screened to determine if there were any segments with significant motion artifacts and were compared alongside accelerometer data also available from the Biostrap cloud server.

For the cross-validation of vasoconstriction parameters, a subset of the control participants wore the ANO sleep device with a finger sensor simultaneously with the Biostrap wristband sensor. Sleepware G3 (version 3.9.7.0; Respironics Inc) provides the capability of storing acquisitions from an ANO sleep device on a cloud-based server and exporting the .edf files (European Data Format) [15]. We converted data from both wristband and finger sensors into .Mat files (MATLAB) to compare them synchronously on 1 platform and derive the physiological parameters of interest.

### PPG Signal Processing and Derivation of Vasoconstriction Parameters

#### Signal Processing

**Figure 1.**
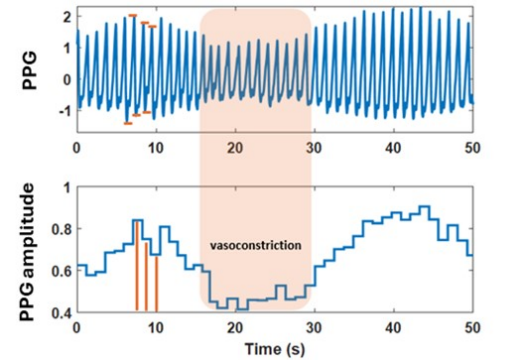
Photoplethysmography (PPG) amplitude calculated from peak-to-trough of each pulse of raw PPG signal with a decrease in amplitude signifying vasoconstriction.

**Figure 2.**
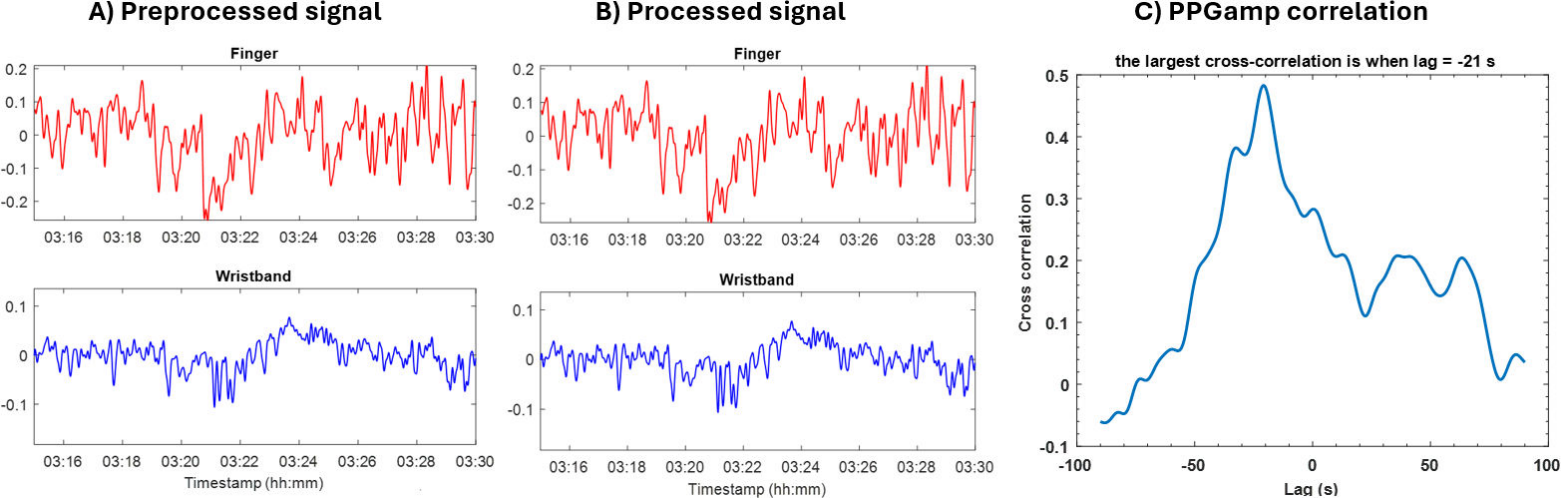
Representative 15-minute PPGamp segments from finger and wristband PPG sensors. (A) Preprocessed signals depicting finger and wristband PPGamp extracted from raw PPG signals. (B) Processed signals depicting “clean” finger and wristband PPGamp after removal of artifacts and respiratory influence. (C) Cross-correlation of the finger and wristband PPGamp segments. In this example, the maximal correlation occurs at a lag of −21 seconds. PPG: photoplethysmography; PPGamp: photoplethysmography amplitude.

The peak-to-trough amplitude of each pulse in the raw PPG signal reflects the pulsatile change in arteriolar blood volume, with a lower amplitude indicating vasoconstriction (Figure 1). The photoplethysmography amplitude (PPGamp), along with the pulse duration, was calculated from both wrist and finger raw PPG signals and divided into consecutive 15-minute segments (Figure 2A). First, segments with clear signal loss were visually identified and manually trimmed or eliminated. Second, outliers were removed by replacing values exceeding the 98th percentile within each segment with the 98th percentile value. Finally, the signal was normalized to its 95th percentile. After linear detrending, the respiratory influence (frequency >0.15 Hz) was filtered out from both wristband and finger PPGamp signals (Figure 2B). These processed “clean” PPGamp signals from each device were used to calculate corresponding vasoconstriction parameters.

Since each device had its own independent time base, the corresponding finger and wristband PPGamp signals had to be aligned in time in order to facilitate comparison of vasoconstriction parameters across devices. The cross-correlation function is a standard method in time series analysis that is used to assess the degree of linear similarity between 2 signals and has been comprehensively described in the literature [16]. We used this technique to compare and align the PPGamp signals from the 2 different devices. The PPGamp time series from each device was first autocorrelated to ensure that these signals represent physiological measurements rather than random noise. Thereafter, corresponding segments of the finger and wristband PPGamp signals underwent multiple time shifts over the other to locate the point of maximum positive cross-correlation within a +30 or –30 second window, thus synchronizing the PPGamp signals from both devices (Figure 2C).

#### Calculation of Vasoconstriction Parameters: Mvasoc and PPGampCV

**Figure 3.**
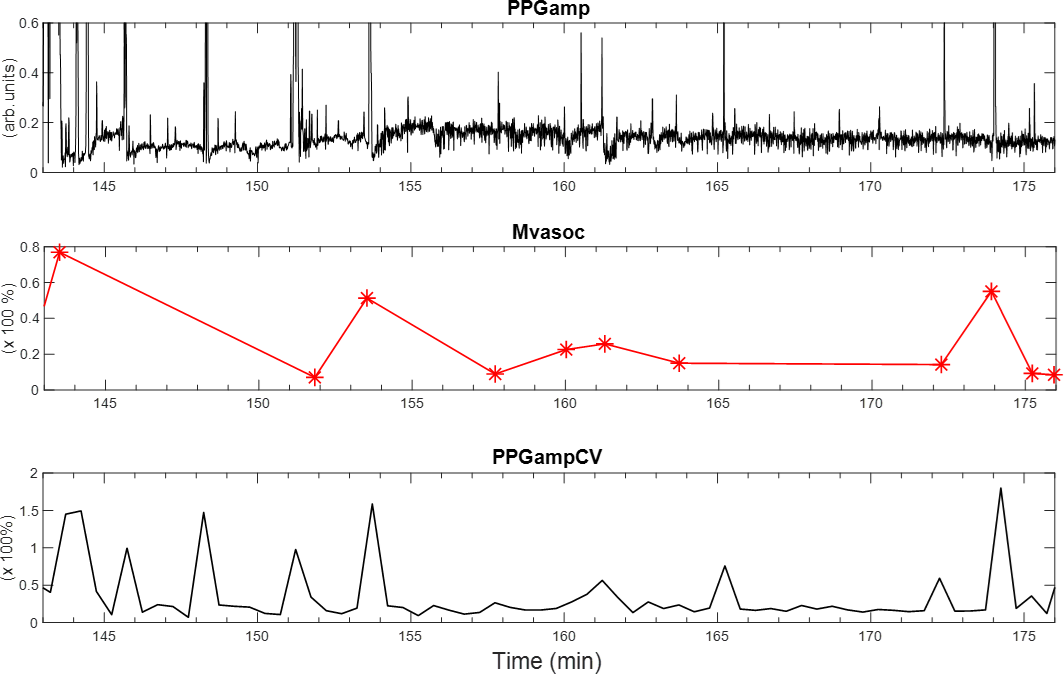
The first row represents a PPGamp segment from wristband sensor, and the next 2 rows show corresponding Mvasoc and PPGampCV parameters derived from the PPGamp. PPGamp: photoplethysmography amplitude; PPGampCV: photoplethysmography amplitude coefficient of variation; Mvasoc: magnitude of vasoconstriction.

We have previously introduced Mvasoc as a unique nocturnal vasoconstriction biomarker that takes into account the frequency, magnitude, and duration of autonomic-mediated spontaneous vasoconstrictions occurring each night [8]. The algorithm was developed to detect and quantify a vasoconstriction event as a significant reduction in PPGamp below the preceding 15-second baseline. Mvasoc is subsequently defined as the ratio of the area of the drop below baseline in PPGamp and the duration of the vasoconstriction, normalized by the baseline PPGamp level (described in detail in the supplement of Chalacheva et al [8]). Because many vasoconstriction events can occur during the night, the median Mvasoc value per night is used as a quantitative index of the user’s nocturnal vasoconstriction behavior during a given night [8]. However, when the Mvasoc algorithm is applied to PPGamp signals, the rule-based measure can be sensitive to time-varying changes in signal-to-noise ratio, and thus the time locations and amplitude of the detected vasoconstrictions can vary across PPG devices. To overcome this limitation, we used a second measure to quantify the dynamic variability in beat-to-beat PPGamp. More specifically, the coefficient of variation (CV; CV=SD/mean) of PPGamp within a sliding time window of 5 minutes was calculated every 30 seconds. In initial studies, this parameter (ie, PPGampCV) correlated closely and robustly with Mvasoc, while simultaneously eliminating the need for a rule-based algorithm to detect “significant vasoconstriction” events. Figure 3 is a representation of Mvasoc and PPGampCV parameters from a wristband PPGamp segment.

#### Within-Device Correlation of Mvasoc and PPGampCV Vasoconstriction Parameters

To rigorously assess whether PPGampCV can be used as a surrogate for Mvasoc, we performed a correlation analysis of the 2 parameters as measured by the fingertip sensor in the ANO device and wrist sensor in the Biostrap device and derived the following within-device correlation coefficient (r_wd_) values:

1. Overnight r_wd_: represents the correlation between vasoconstriction parameters Mvasoc and PPGampCV for the whole night
2. Peak r_wd_: represents the segment with the maximum correlation of Mvasoc and PPGampCV out of all the 15-minute segments on a given night

#### Across-Device Cross-Validation of Vasoconstriction Parameter

To determine if the wrist device gave the same results as the fingertip sensor, we correlated the PPGampCV parameter from the wristband and “gold-standard” finger sensors worn at the same time. The corresponding 15-minute segments from both devices were only considered for cross-correlation if at least 3 vasoconstriction events were detected within each segment. We correlated the PPGampCV between the finger and wrist PPGamp each night and derived the following across-device correlation coefficient (r_ad_) values:

1. Overnight r_ad_: represents the correlation of PPGampCV across wrist and finger sensor for all 15-minute segments per night
2. Peak r_ad_: represents the segment with the maximal correlation of PPGampCV out of all corresponding 15-minute segments per night.

In all the analyses mentioned earlier, *P* values of <.05 suggested that the correlation between signals was statistically significant.

## Results

### Longitudinal Monitoring of Vasoconstriction Parameters With Wristband Device

A total of 12 patients with SCD and 6 control participants participated in the study. The mean age of the sample was 25 (range 14‐40) years. The mean hemoglobin was 8.6 g/dl (SD 1.9 g/dl) and 14.1 g/dl (SD 1.8 g/dl) for patients with SCD and controls, respectively. A total of 10 (83%) of the 12 patients with SCD reported the use of disease-modifying therapy with hydroxyurea at the time of the study. A total of 131 nocturnal PPG recordings were made with wristband devices in all participants. Patients with SCD had a total of 79 (60%) nocturnal recordings (range 1‐15 nights), and controls had 52 (40%) nocturnal recordings (range 1‐19 nights). While participants wore the wristband for a variable number of nights due to device availability, there were no reported logistical issues with functioning of the device, and participants reported ease of use.

**Figure 4.**
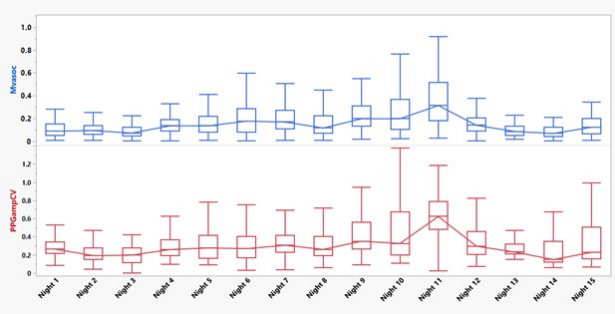
Longitudinal monitoring of vasoconstriction parameters Mvasoc and PPGampCV in a patient with sickle cell disease from the wristband device. The box plot shows the median and IQR per night, and the midline shows the median of these parameters across all nights. PPGampCV: photoplethysmography amplitude coefficient of variation; Mvasoc: magnitude of vasoconstriction.

Longitudinal measurements of Mvasoc and PPGampCV vasoconstriction parameters showed significant variability within each night and from night to night. A representative participant’s longitudinal data are depicted in Figure 4 and show that there is a wider range of vasoconstriction activity on nights 10 and 11, with an increase in the median Mvasoc and PPGampCV noted on those nights, reflecting increased autonomic vasomotor activity. On visual inspection, the night-to-night changes in Mvasoc and PPGampCV appeared to closely track each other, suggesting a potential correlation between the 2 parameters.

**Figure 5.**
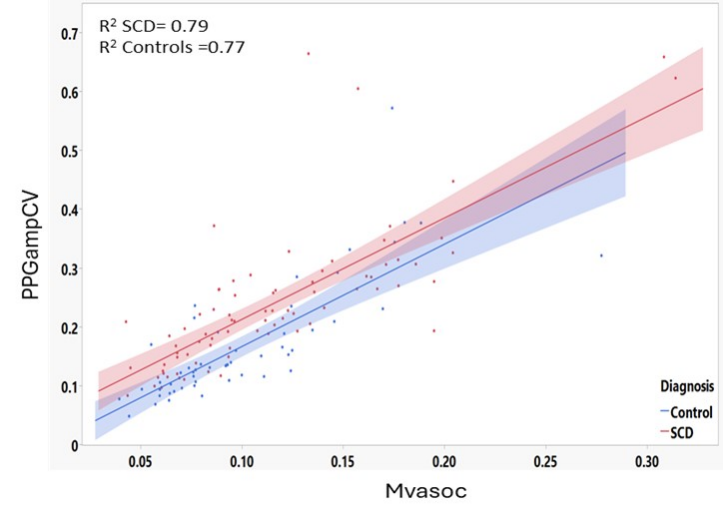
Correlation of median Mvasoc with corresponding median PPGampCV for all recorded nights from the wristband device among patients with SCD and controls. Mvasoc: magnitude of vasoconstriction; PPGampCV: photoplethysmography amplitude coefficient of variation; SCD: sickle cell disease.

Indeed, the median Mvasoc of each night was closely correlated with the corresponding median PPGampCV both for patients with SCD and controls in all the nightly data (patients with SCD: *R*^2^=0.79; controls: *R*^2^=0.77; Figure 5).

### Within-Device Correlation of Vasoconstriction Parameters (Mvasoc and PPGampCV)

While median Mvasoc and PPGampCV track closely in longitudinal nightly recordings, we further analyzed the correlation of these parameters during time segments within individual nightly recordings. Three control participants wore both finger and wristband PPG devices for a total of 9 nights, and each nighttime recording was divided into 15-minute segments. Table 1 shows the number of vasoconstriction (Mvasoc) events detected each night from both the finger sensor and wristband devices. There were a total of 113 viable segments over 9 nights, with an average of 10 and 11 vasoconstriction events detected during each segment from the finger and wristband sensors, respectively.

**Table 1.** Number of vasoconstriction events (magnitude of vasoconstriction) detected each night from the finger and wristband sensors.

	Finger sensor, n	Wristband, n
Participant 1		
Night 1 (5 segments)	34	56
Night 2 (8 segments)	90	104
Night 3 (12 segments)	140	152
Participant 2		
Night 1 (20 segments)	186	226
Night 2 (18 segments)	175	195
Night 3 (8 segments)	71	120
Participant 3		
Night 1 (19 segments)	206	214
Night 2 (18 segments)	200	190
Night 3 (5 segments)	40	57

**Figure 6.**
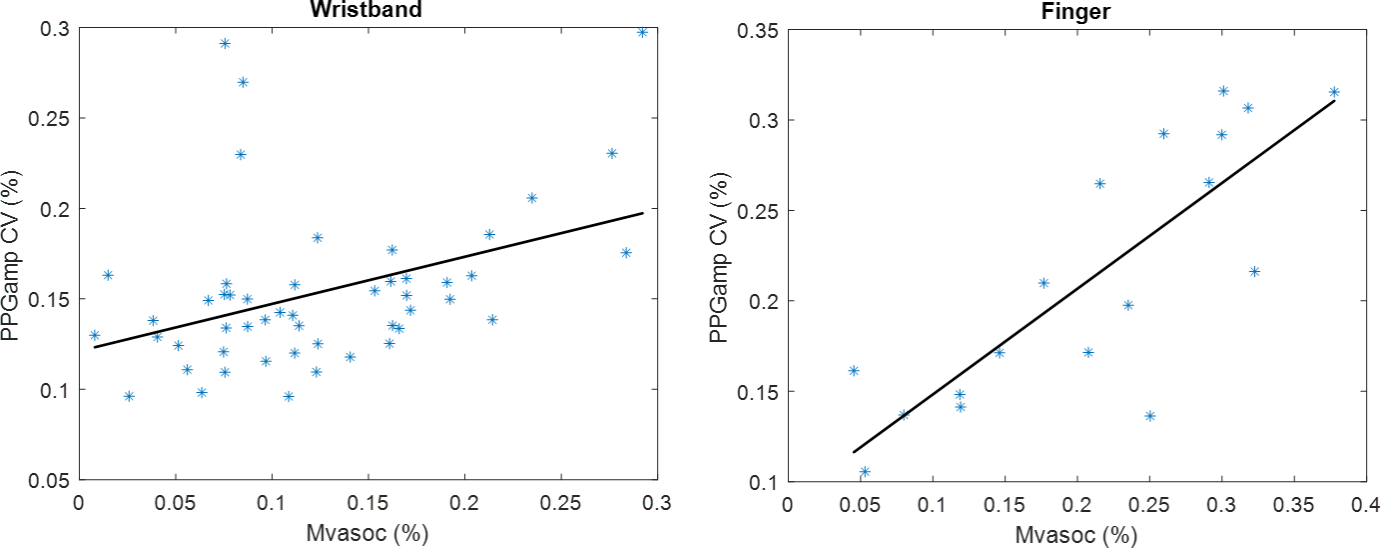
Example of overnight correlation (r_wd_) between vasoconstriction parameters, Mvasoc and PPGampCV, for 1 control 1 night in wristband (Biostrap) and finger sensor (AliceNightOne). Panel (a wristband*; r*=0.40; *P*<.005) and AliceNightOne device in panel (b finger sensor; *r*=0.82; *P*<.0001). The significant correlation between Mvasoc and PPGampCV assured us that PPGampCV can be used as a surrogate marker of the magnitude of vasoconstriction. Mvasoc: magnitude of vasoconstriction; PPGampCV: photoplethysmography amplitude coefficient of variation.

Cross-correlation of Mvasoc and corresponding PPGampCV values were calculated for each segment of these 9 nights. Overnight r_wd_ correlation and segmental peak r_wd_ correlation coefficient data for all 9 nights (Table 2) show that Mvasoc was significantly correlated with PPGampCV each night within both devices and within almost all segments. The highest overnight r_wd_ correlation was 0.82 (*P*<.001) for the finger sensor and 0.69 (*P*<.001) for the wristband for all nights. The highest peak r_wd_ correlation was 0.99 (*P*<.05) in both devices for all nights (113 segments). Figure 6 shows the within-device overnight r_wd_ correlation between Mvasoc and PPGampCV for a representative night.

**Table 2.** Cross-correlation between magnitude of vasoconstriction (Mvasoc) and photoplethysmography amplitude coefficient of variation (PPGampCV) using overnight correlation coefficient (overnight r_wd_) and peak correlation coefficient (peak r_wd_) within wristband (Biostrap) and finger sensors (AliceNightOne).

Mvasoc versus PPGampCV correlation		Finger sensor	Wristband
Participant 1			
Night 1 (5 segments)			
Overnight r_wd_^a^		0.82	0.40
Peak r_wd_^b^		0.90	0.74
Night 2 (8 segments)			
Overnight r_wd_		0.35	0.47
Peak r_wd_		0.93	0.78
Night 3 (12 segments)			
Overnight r_wd_		0.51	0.41
Peak r_wd_		0.82	0.80
Participant 2			
Night 1 (20 segments)			
Overnight r_wd_		0.32	0.69
Peak r_wd_		0.91	0.96
Night 2 (18 segments)			
Overnight r_wd_		0.29	0.48
Peak r_wd_		0.97	0.93
Night 3 (8 segments)			
Overnight r_wd_		0.47	0.34
Peak r_wd_		0.99	0.92
Participant 3			
Night 1 (19 segments)			
Overnight r_wd_		0.40	0.49
Peak r_wd_		0.80	0.99
Night 2 (18 segments)			
Overnight r_wd_		0.37	0.51
Peak r_wd_		0.79	0.95
Night 3 (5 segments)			
Overnight r_wd_		0.33	0.30
Peak r_wd_		0.95	0.50^c^

a Overnight r_wd_**:** correlation coefficient value of vasoconstriction parameters, Mvasoc and PPGampCV, for the whole night; *P*<.05.

b Peak r_wd_**:** correlation coefficient value of segment with highest correlation out of all 15-minute segments per night; *P*<.05.

c *P*>.05.

### Across-Device Cross-Validation of Vasoconstriction Parameters (CV)

**Figure 7.**
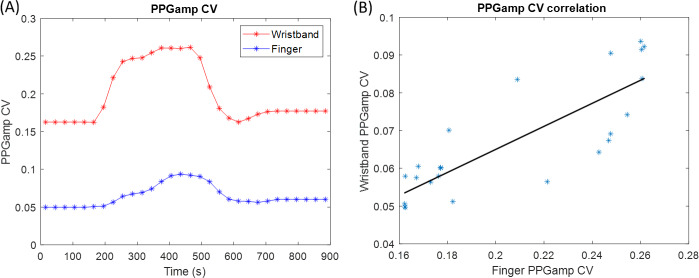
Correlation of PPGampCV between the wristband and finger device for a representative 15-minute segment of 1 night. The left panel (A) shows the corresponding PPGampCV values from both devices over time, and the right panel (B) shows the correlation of PPGampCV values between the 2 devices (*r*=0.84; *P*<.001). PPGampCV: photoplethysmography amplitude coefficient of variation.

Since the PPGampCV serves as a surrogate marker of vasoconstriction and is overall easier to derive from the PPG and compare across devices, we chose the PPGampCV to perform cross-validation of vasoconstriction parameters between the 2 devices. The highest overnight r_ad_ cross-correlation for all nights was 0.70, and the highest peak r_ad_ cross-correlation for all nights was 0.99. Table 3 shows the PPGampCV overnight correlation (r_ad_) coefficient and segmental peak (r_ad_) correlation coefficient data for all 9 nights (123 segments). Figure 7 shows an example of correlation analysis of PPGampCV across devices in 1 corresponding 15-minute segment.

**Table 3.** Overnight r_ad_ correlation and peak r_ad_ correlation coefficients of photoplethysmography amplitude coefficient of variation between wristband (Biostrap) and finger sensor (AliceNightOne).

	Overnight r_ad_^a^	Peak r_ad_^b^
Participant 1		
Night 1 (5 segments)	0.24	0.76
Night 2 (8 segments)	0.50	0.90
Night 3 (12 segments)	0.70	0.89
Participant 2		
Night 1 (20 segments)	0.49	0.97
Night 2 (18 segments)	0.57	0.99
Night 3 (8 segments)	0.52	0.96
Participant 3		
Night 1 (23 segments)	0.36	0.97
Night 2 (24 segments)	0.62	0.96
Night 3 (5 segments)	0.65	0.56

a Overnight r_ad_: represents the correlation coefficient value for all 15-minute segments per night;* P*<.05.

b Peak r_ad_: represents the correlation coefficient value of segment with highest correlation out of all 15-minute segments per night; *P*<.05.

## Discussion

### Principal Findings

The sudden, unpredictable onset of VOC remains the major cause of morbidity and the leading cause of hospitalizations in patients with SCD [17]. While some patients experience an aura or premonition that a VOC is about to occur [18], there are no objective biomarkers that can predict the risk of an imminent VOC and thus open up a therapeutic window to abort its propagation. We have introduced nocturnal vasoconstriction indices of autonomic peripheral vasoreactivity that have the potential to be predictive biomarkers of impending VOC crisis. This study establishes the feasibility of measuring these biomarkers in a real-world setting with a wearable device, such that the vasoconstriction biomarker can be validated in larger populations. To the best of our knowledge, this is the first study that uses the PPG signal from a wearable-based sensor system to quantify microvascular blood flow changes accompanying peripheral vasoconstriction.

We successfully used the Biostrap wearable wristband device to longitudinally monitor raw PPG signals and derive autonomic vasoconstriction parameters. Participants reported ease of use, and no significant barriers were identified in using the device. There was substantial night-to-night variability in the median Mvasoc and PPGampCV parameters within participants, which implies varying levels of sympathetic vasoreactivity. This has potential implications for tracking pain and imminent VOC risk, as SCD physiology dictates that vasoconstriction and resultant decrease in microvascular perfusion increase the likelihood of vaso-occlusion.

Although Mvasoc is a direct quantification of vasoconstriction events from the PPGamp signal, we found that this parameter is more prone to artifacts that can plague wearable device signals. The PPGampCV is a more robust measure of variability of the PPGamp amplitude that closely reflects Mvasoc and is an acceptable surrogate for monitoring vasoconstriction. The ANO with a fingertip PPG sensor is a widely used Food and Drug Administration–approved home sleep device that represents a reduced version of the clinical polysomnography. The vasoconstrictions measured at the fingertip are generally stronger than the corresponding events measured at the wrist, and thus the signal-to-noise ratio of the fingertip signal is higher than that of the wrist signal. Physiologically, this is to be expected since the fingers are highly innervated by alpha-adrenergic fibers and are also highly vascularized along with large numbers of arteriovenous anastomoses, compared to the wrist. This results in much faster and stronger vasoconstrictive responses in the fingers vis-à-vis the wrist. These differences in perfusion also have the potential to impact the cross-correlation values of the PPGampCV signals between the fingertip and the wrist. Using a cutoff of ≥0.5 as an acceptable correlation coefficient, we still see that there is mostly moderate to very strong correlation of PPGampCV between ANO and Biostrap. This shows that the wristband performs comparably to the “gold-standard” fingertip PPG while being much more practical to use. The overall correlation of PPGampCV for each night also remained statistically significant and acceptable between devices, suggesting that the wristband device remains a viable and feasible tool to deploy for longer-term, easy-to-use remote longitudinal monitoring of autonomic vasoreactivity.

This study is also unique in the sense that most wearable devices that purport to provide information about autonomic function focus on heart rate variability and SpO2 changes, but do not monitor changes in PPG amplitude, indicative of peripheral vasoconstriction [9]. Our Mvasoc algorithm and PPGampCV biomarker can be adapted to any wearable device that uses the PPG signal to measure ANS activity and quantify vasoconstriction in users with and without SCD. Promising preliminary data suggest a correlation between the Mvasoc parameter and the intensity of daily pain in patients with SCD. In 10 patients with SCD from this same cohort, we successfully tracked daily pain data using a SMS text message–based red cap survey, along with wristband recordings. Presence of pain and intensity of pain were tracked on a scale of 1 to 10. Of the 68 nocturnal wristband recordings with concurrent pain diary entries, 25 days of pain were reported with a pain intensity ranging from 3 to 8. While the data were not powered toward the prediction of a VOC crisis given the limited number of nights of autonomic vasoreactivity monitoring, we found that a higher vasoconstriction index (Mvasoc) and a greater parasympathetic withdrawal in the heart rate variability patterns predicted greater intensity of pain the next day on multivariate regression analysis [19]. While these data hint at possible temporal associations between autonomic vasoreactivity patterns and pain, they need to be validated with longer-term longitudinal monitoring of vasoconstriction parameters in larger patient populations with SCD in order to develop it as a predictive tool for imminent VOC.

In conclusion, our pilot data show that the implementation of PPG from wearable sensors has a high potential for the development of an autonomic vasoreactivity biomarker for VOC in SCD. The portability, cost-effectiveness, and low maintenance of these wearable sensors can make them suitable for remote use. Moreover, these vasoconstriction parameters derived from PPG amplitude can serve as easily accessible and readily quantifiable biomarkers of tissue perfusion that could be valuable in other disease states, such as migraine and coronary artery disease, where vasoreactivity plays a role in disease pathology.

### Limitations

The small sample size of nocturnal recordings, especially with the ANO device, makes it difficult to generalize the results. About 20% of the PPG signal from the nightly recordings was further eliminated due to loss of signal, primarily from the finger sensor. However, the high number of vasoconstriction events detected within each night of the available data adequately powers the correlation analyses and improves the robustness of the data. Both devices were not synchronized on the same time scale; therefore, we attempted to perform cross-correlation to 15-minute segments within a +30 or –30 second window. This technique can result in segment selection errors and lower correlation values. Future studies with concurrent use of the wristband device during clinical sleep studies with finger PPG sensors can be used to validate these results in a larger population. While we have shared preliminary results on the relationship between Mvasoc and daily pain intensity, the study is primarily focused on the ability to detect vasoconstriction events with wearable devices, and we were unable to derive any clinical inference on any causal relationship with VOC onset, the impact of hemoglobin levels, or the use of hydroxyurea on vasoreactivity.

## Abbreviations

ANO: AliceNightOne
ANS: autonomic nervous system
Mvasoc: magnitude of vasoconstriction
PPG: photoplethysmography
PPGamp: PPG amplitude
PPGampCV: PPGamp coefficient of variation
SCD: sickle cell disease
SpO2: oxygen saturation
VOC: vaso-occlusive crises
